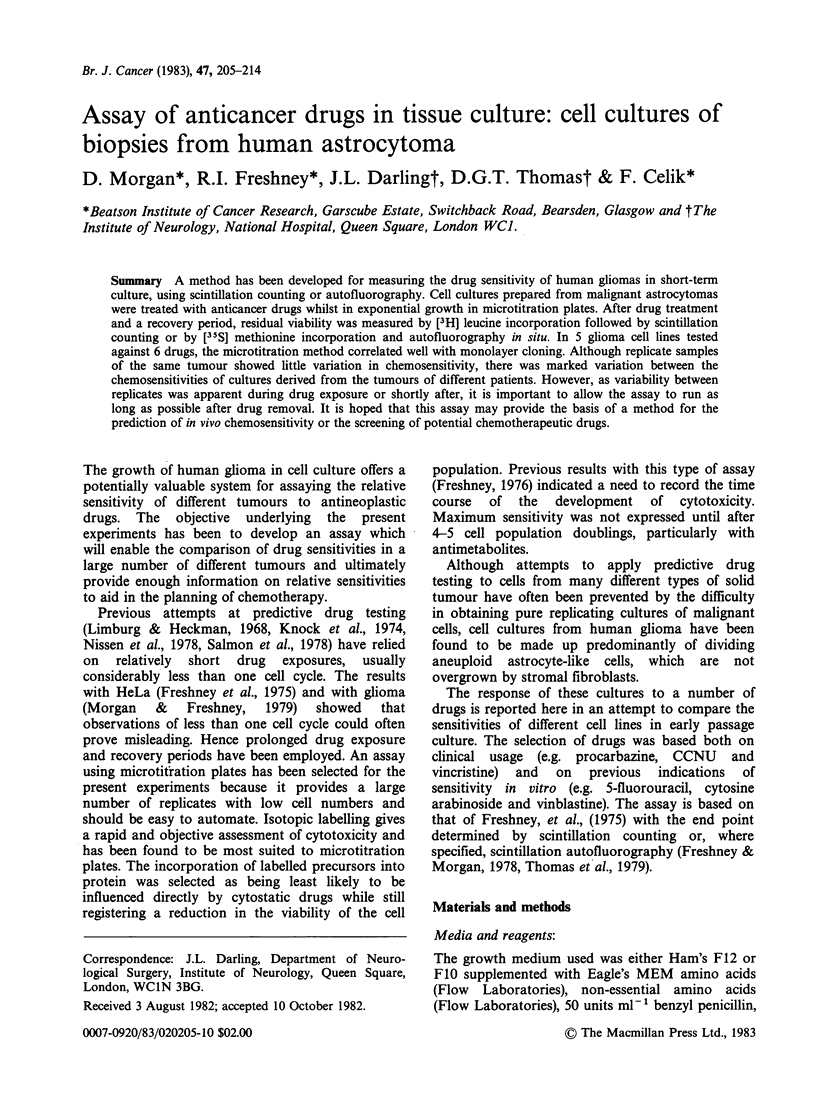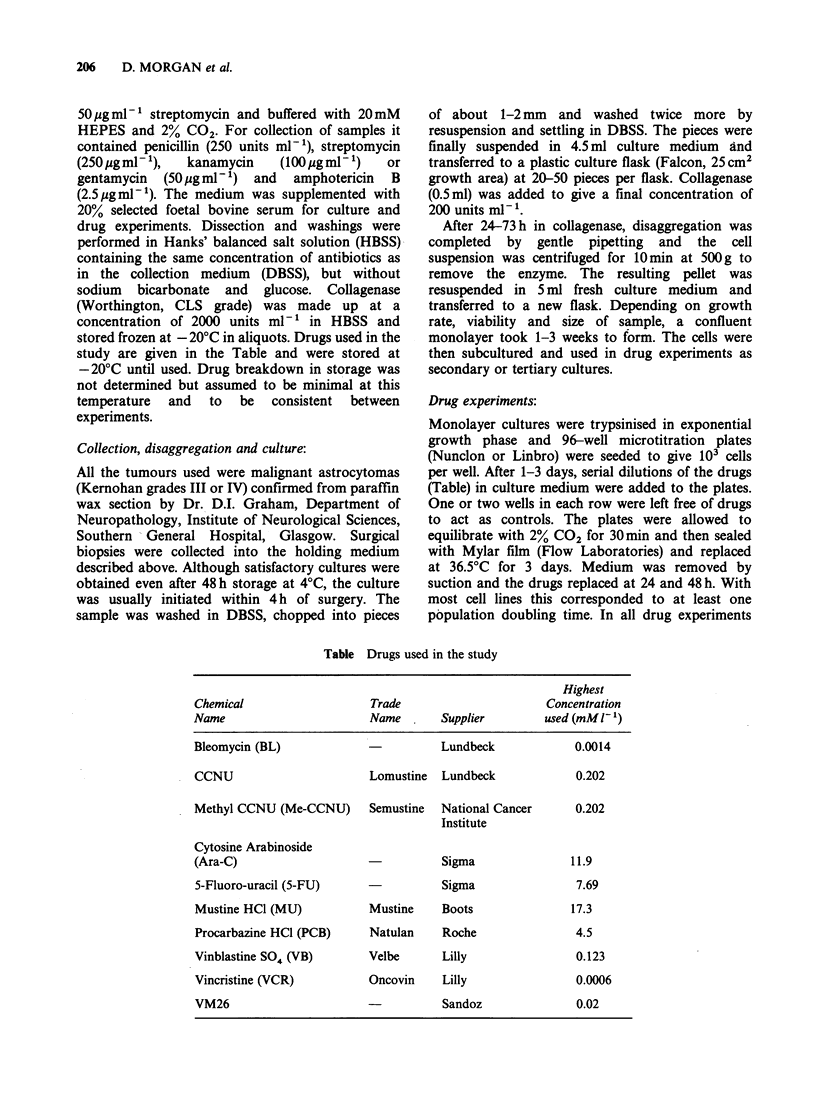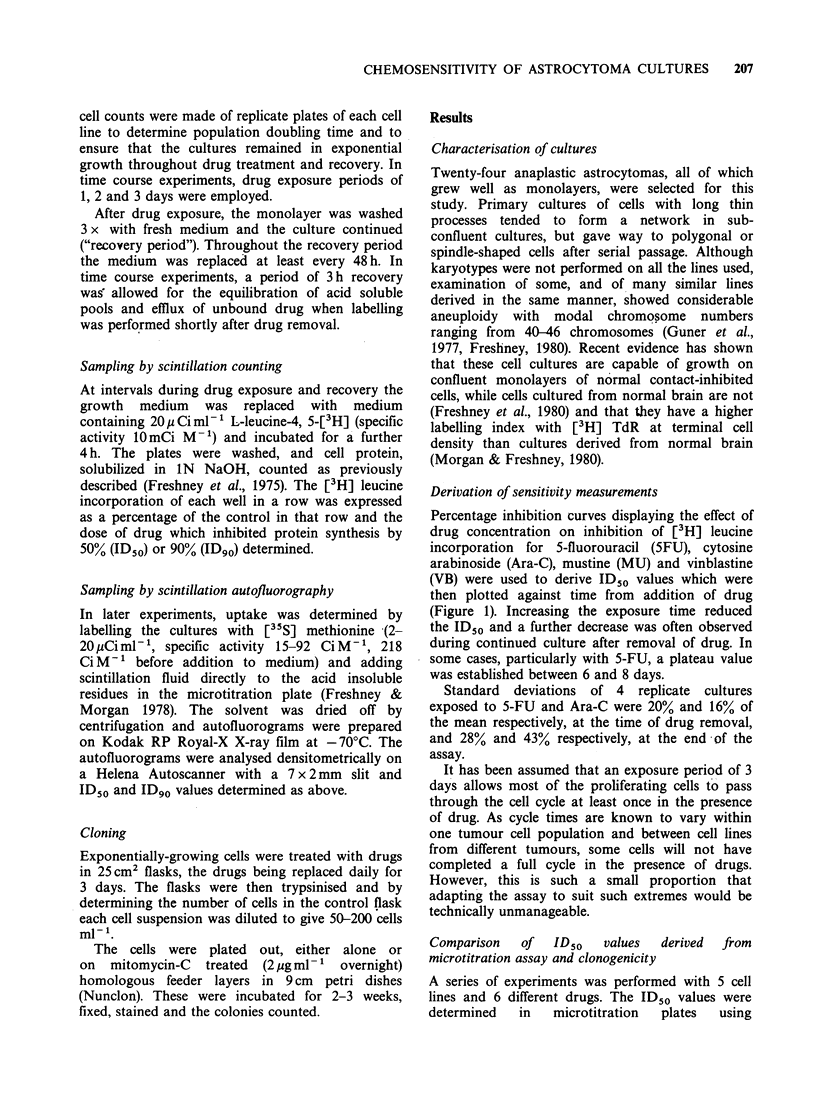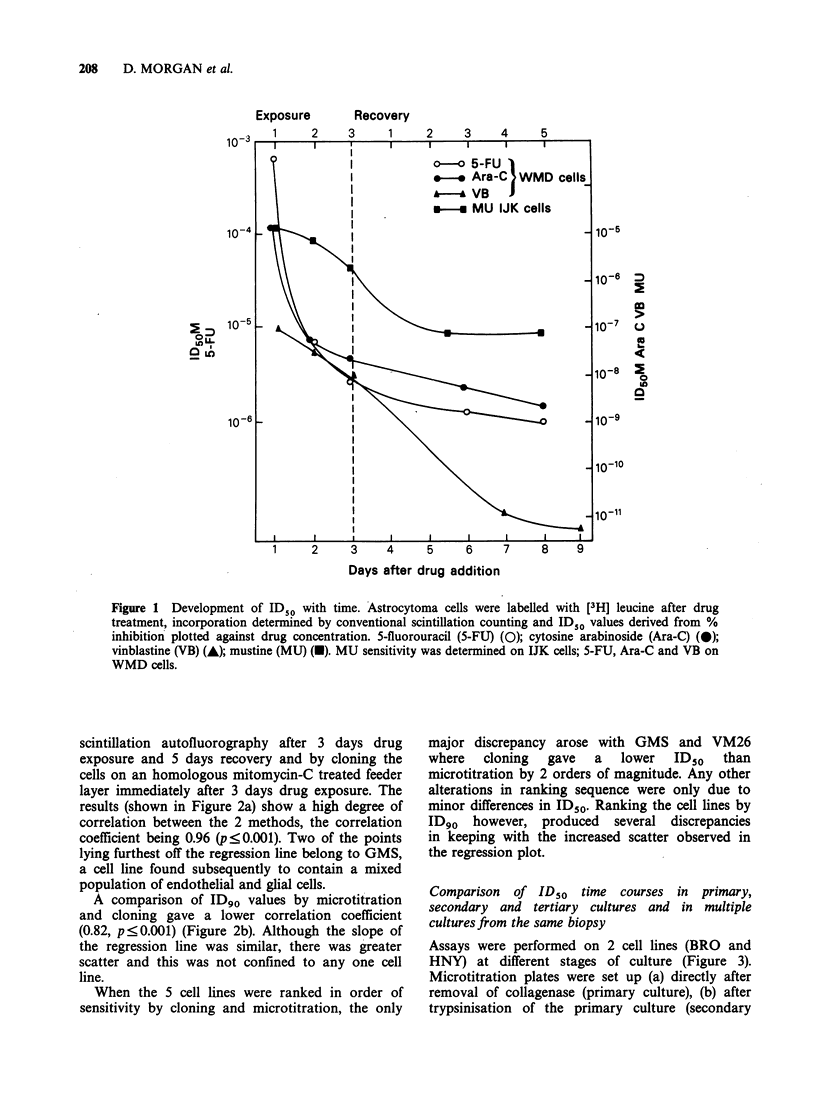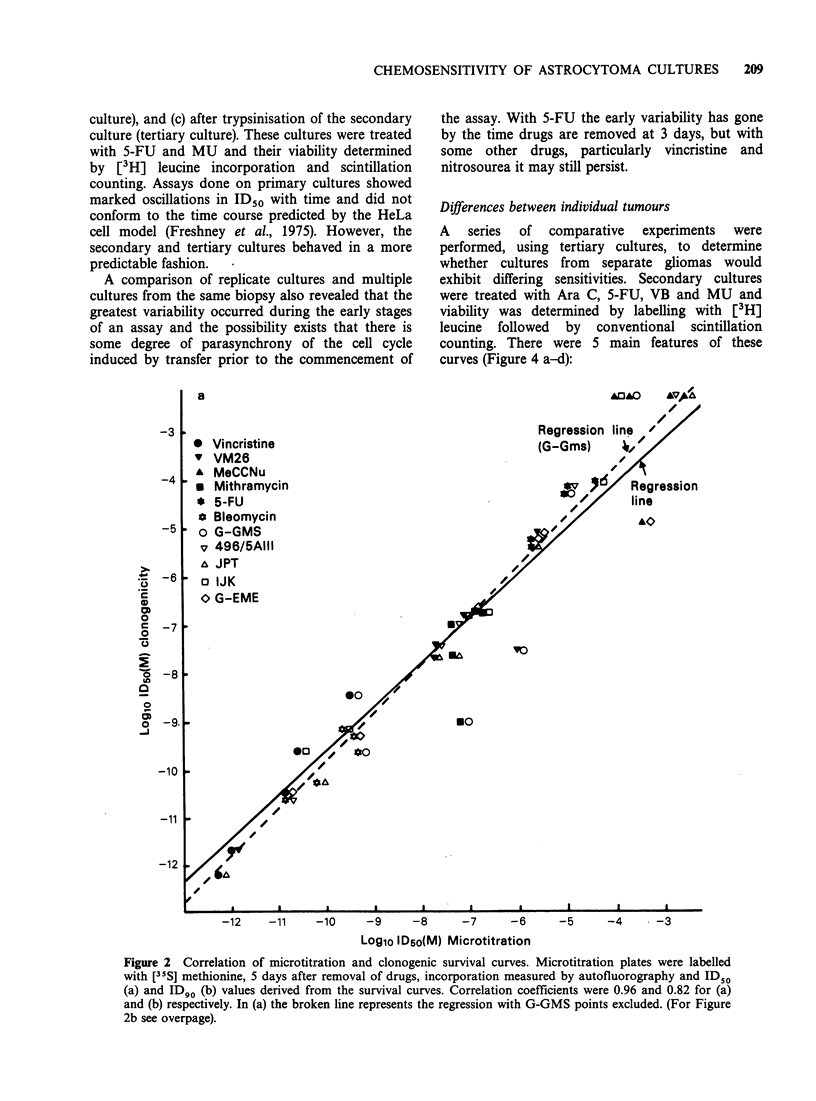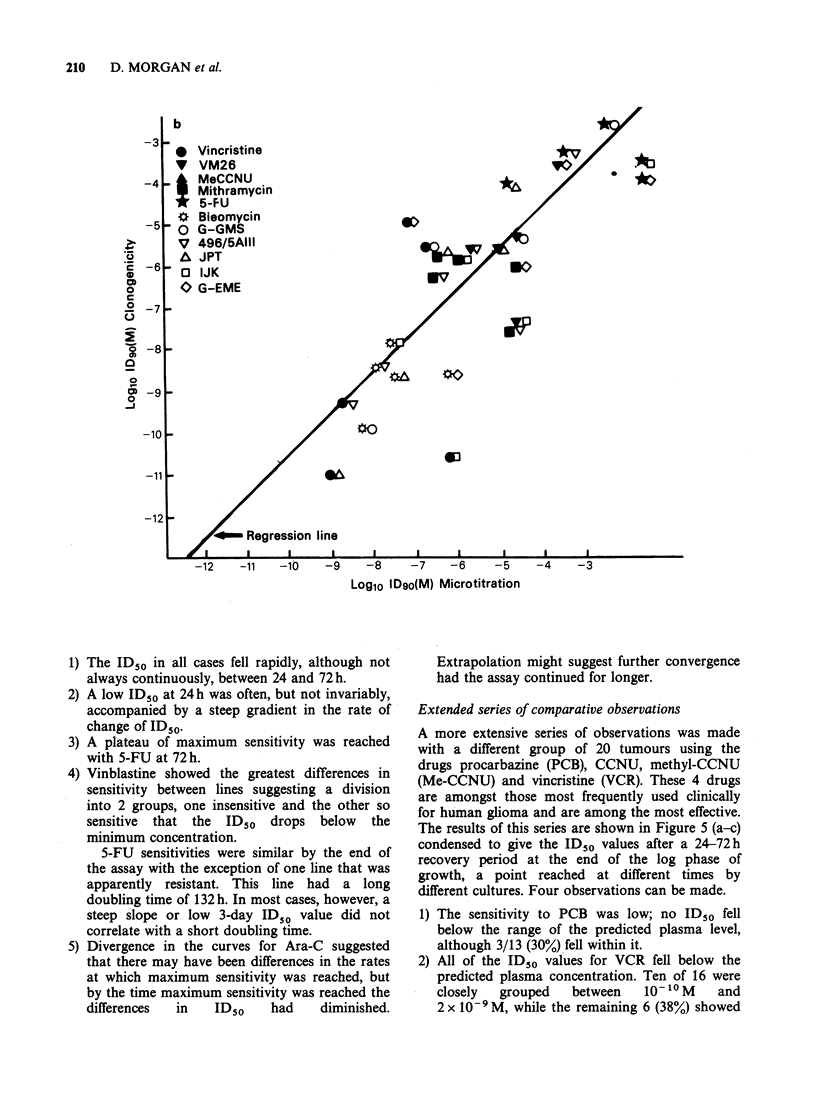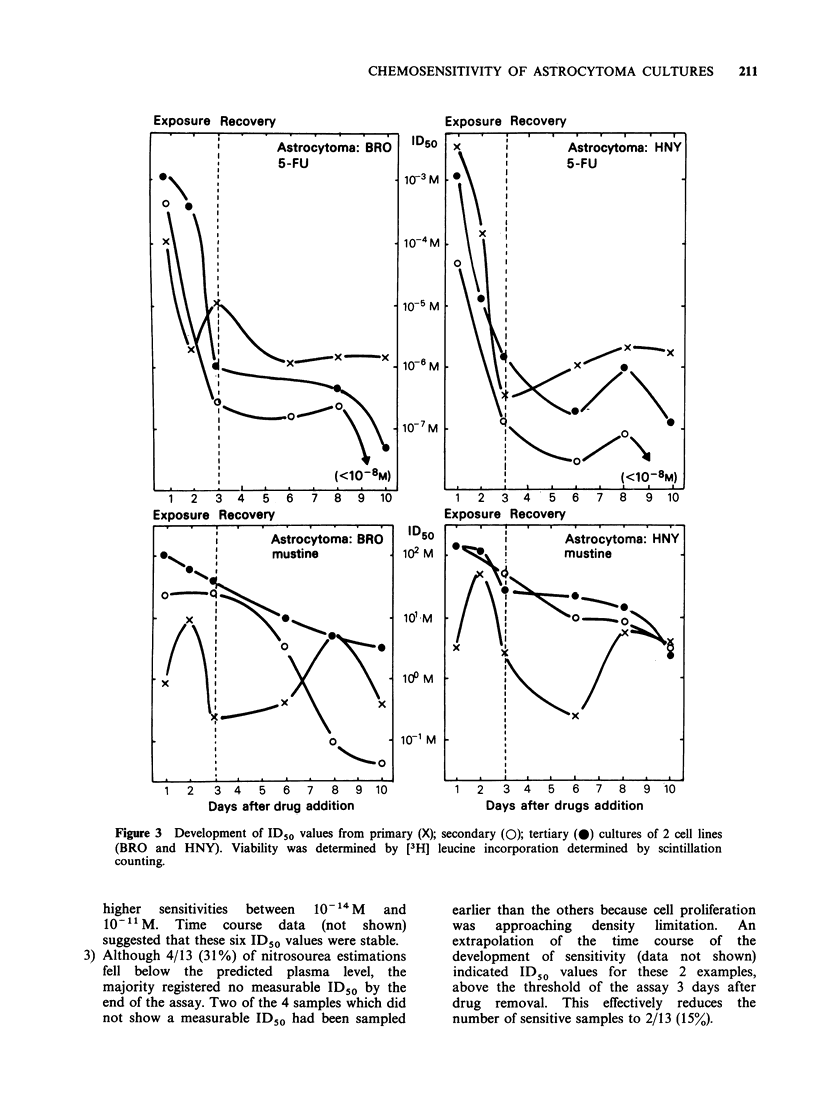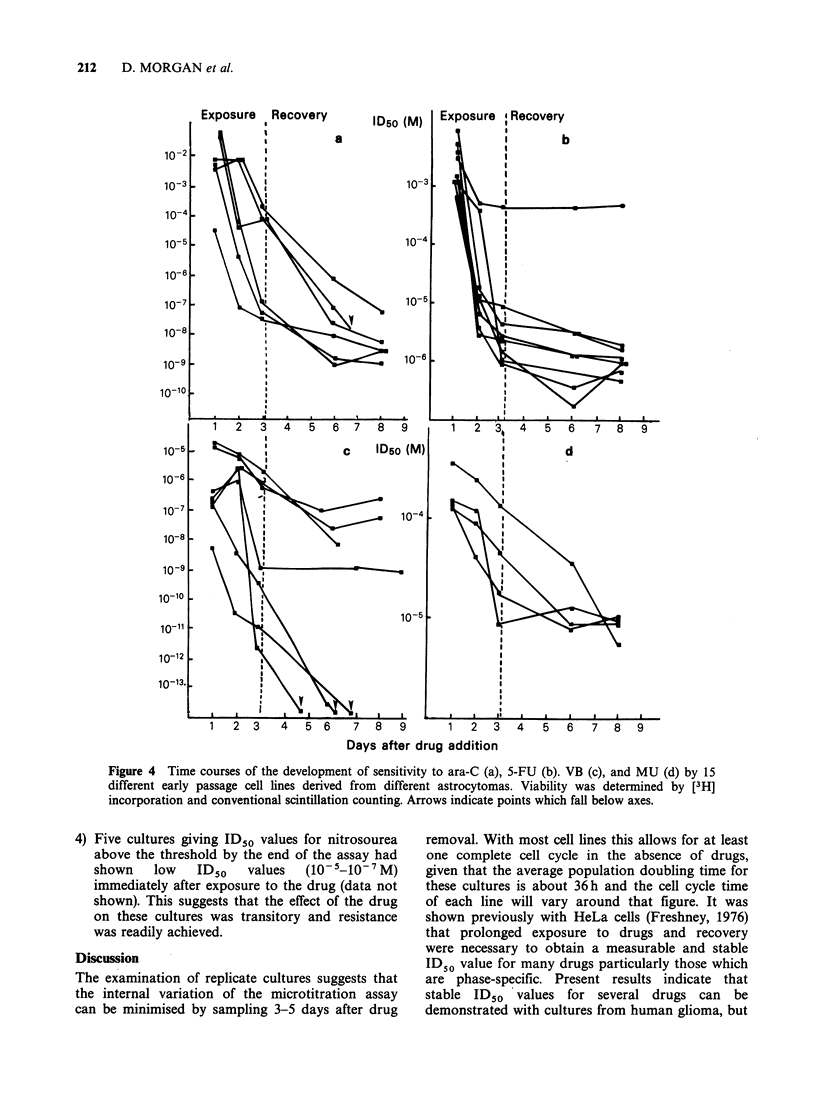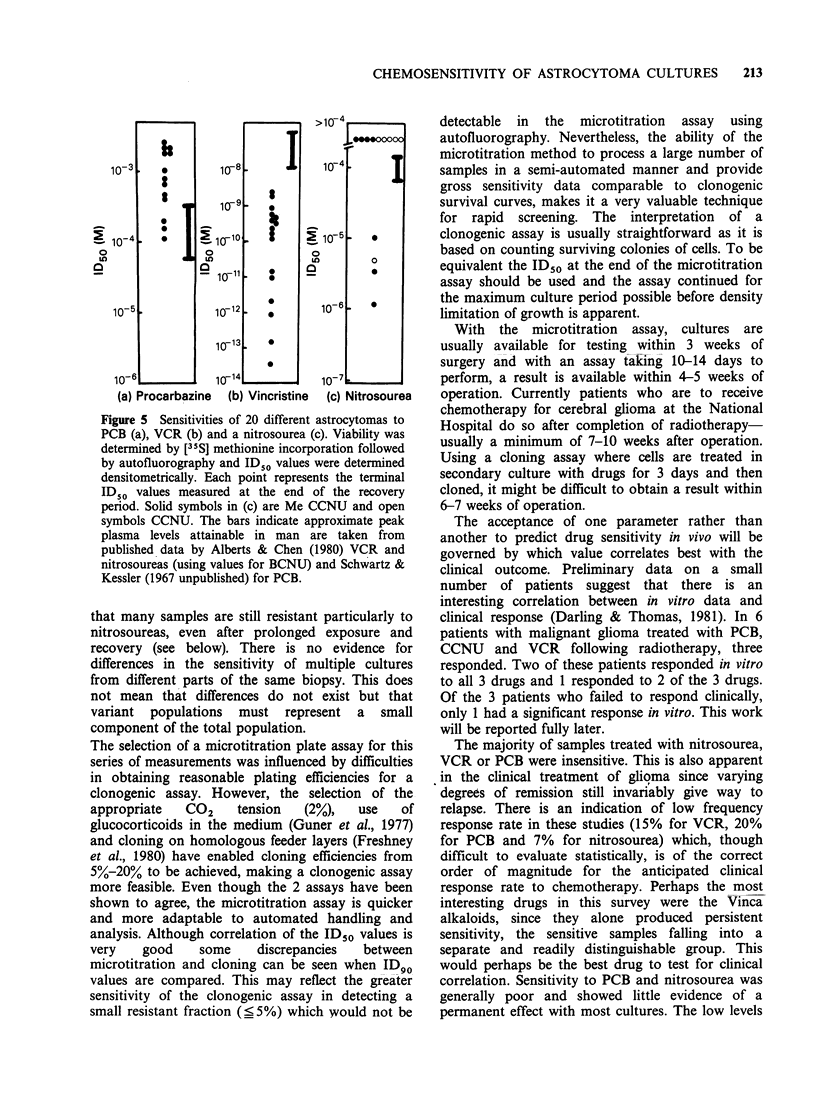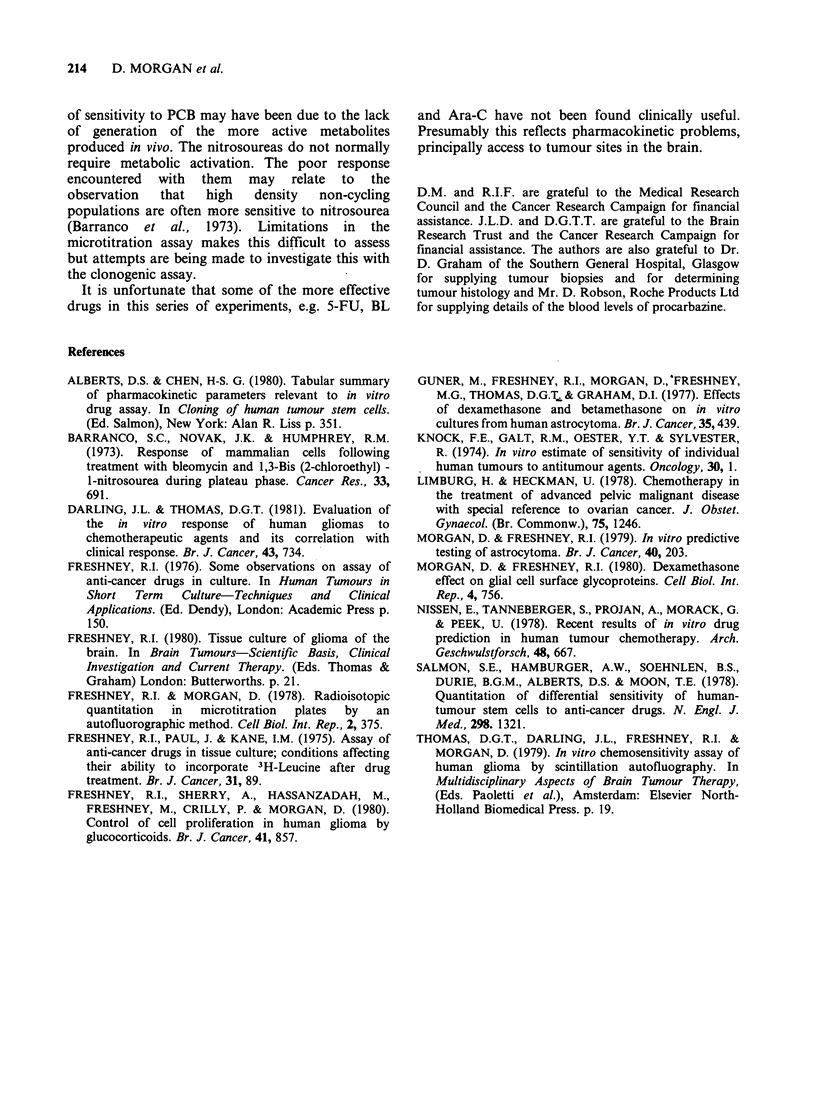# Assay of anticancer drugs in tissue culture: cell cultures of biopsies from human astrocytoma.

**DOI:** 10.1038/bjc.1983.28

**Published:** 1983-02

**Authors:** D. Morgan, R. I. Freshney, J. L. Darling, D. G. Thomas, F. Celik

## Abstract

A method has been developed for measuring the drug sensitivity of human gliomas in short-term culture, using scintillation counting or autofluorography. Cell cultures prepared from malignant astrocytomas were treated with anticancer drugs whilst in exponential growth in microtitration plates. After drug treatment and a recovery period, residual viability was measured by [3H] leucine incorporation followed by scintillation counting or by [35S] methionine incorporation and autofluorography in situ. In 5 glioma cell lines tested against 6 drugs, the microtitration method correlated well with monolayer cloning. Although replicate samples of the same tumour showed little variation in chemosensitivity, there was marked variation between the chemosensitivities of cultures derived from the tumours of different patients. However, as variability between replicates was apparent during drug exposure or shortly after, it is important to allow the assay to run as long as possible after drug removal. It is hoped that this assay may provide the basis of a method for the prediction of in vivo chemosensitivity or the screening of potential chemotherapeutic drugs.


Br. J. Cancer (1983), 47, 205–214

### Assay of anticancer drugs in tissue culture: cell cultures of biopsies from human astrocytoma

D. Morgan*, R.I. Freshney*, J.L. Darling†, D.G.T. Thomas† & F. Celik*

*Beatson Institute of Cancer Research, Garscube Estate, Switchback Road, Bearsden, Glasgow and †The Institute of Neurology, National Hospital, Queen Square, London WC1.

**Summary** A method has been developed for measuring the drug sensitivity of human gliomas in short-term culture, using scintillation counting or autofluorography. Cell cultures prepared from malignant astrocytomas were treated with anticancer drugs whilst in exponential growth in microtitration plates. After drug treatment and a recovery period, residual viability was measured by [$^3$H] leucine incorporation followed by scintillation counting or by [$^{35}$S] methionine incorporation and autofluorography *in situ*. In 5 glioma cell lines tested against 6 drugs, the microtitration method correlated well with monolayer cloning. Although replicate samples of the same tumour showed little variation in chemosensitivity, there was marked variation between the chemosensitivities of cultures derived from the tumours of different patients. However, as variability between replicates was apparent during drug exposure or shortly after, it is important to allow the assay to run as long as possible after drug removal. It is hoped that this assay may provide the basis of a method for the prediction of *in vivo* chemosensitivity or the screening of potential chemotherapeutic drugs.

The growth of human glioma in cell culture offers a potentially valuable system for assaying the relative sensitivity of different tumours to antineoplastic drugs. The objective underlying the present experiments has been to develop an assay which will enable the comparison of drug sensitivities in a large number of different tumours and ultimately provide enough information on relative sensitivities to aid in the planning of chemotherapy.

Previous attempts at predictive drug testing (Limburg & Heckman, 1968, Knock *et al.*, 1974, Nissen *et al.*, 1978, Salmon *et al.*, 1978) have relied on relatively short drug exposures, usually considerably less than one cell cycle. The results with HeLa (Freshney *et al.*, 1975) and with glioma (Morgan & Freshney, 1979) showed that observations of less than one cell cycle could often prove misleading. Hence prolonged drug exposure and recovery periods have been employed. An assay using microtitration plates has been selected for the present experiments because it provides a large number of replicates with low cell numbers and should be easy to automate. Isotopic labelling gives a rapid and objective assessment of cytotoxicity and has been found to be most suited to microtitration plates. The incorporation of labelled precursors into protein was selected as being least likely to be influenced directly by cytostatic drugs while still registering a reduction in the viability of the cell population. Previous results with this type of assay (Freshney, 1976) indicated a need to record the time course of the development of cytotoxicity. Maximum sensitivity was not expressed until after 4–5 cell population doublings, particularly with antimetabolites.

Although attempts to apply predictive drug testing to cells from many different types of solid tumour have often been prevented by the difficulty in obtaining pure replicating cultures of malignant cells, cell cultures from human glioma have been found to be made up predominantly of dividing aneuploid astrocyte-like cells, which are not overgrown by stromal fibroblasts.

The response of these cultures to a number of drugs is reported here in an attempt to compare the sensitivities of different cell lines in early passage culture. The selection of drugs was based both on clinical usage (e.g. procarbazine, CCNU and vincristine) and on previous indications of sensitivity *in vitro* (e.g. 5-fluorouracil, cytosine arabinoside and vinblastine). The assay is based on that of Freshney, *et al.*, (1975) with the end point determined by scintillation counting or, where specified, scintillation autofluorography (Freshney & Morgan, 1978, Thomas *et al.*, 1979).

### Materials and methods

*Media and reagents*:

The growth medium used was either Ham's F12 or F10 supplemented with Eagle's MEM amino acids (Flow Laboratories), non-essential amino acids (Flow Laboratories), 50 units ml$^{-1}$ benzyl penicillin,

Correspondence: J.L. Darling, Department of Neurological Surgery, Institute of Neurology, Queen Square, London, WC1N 3BG.

Received 3 August 1982; accepted 10 October 1982.

0007-0920/83/020205-10 $02.00

© The Macmillan Press Ltd., 1983

50 $\mu g\, ml^{-1}$ streptomycin and buffered with 20 mM HEPES and 2% $CO_2$. For collection of samples it contained penicillin (250 units $ml^{-1}$), streptomycin (250 $\mu g\, ml^{-1}$), kanamycin (100 $\mu g\, ml^{-1}$) or gentamycin (50 $\mu g\, ml^{-1}$) and amphotericin B (2.5 $\mu g\, ml^{-1}$). The medium was supplemented with 20% selected foetal bovine serum for culture and drug experiments. Dissection and washings were performed in Hanks' balanced salt solution (HBSS) containing the same concentration of antibiotics as in the collection medium (DBSS), but without sodium bicarbonate and glucose. Collagenase (Worthington, CLS grade) was made up at a concentration of 2000 units $ml^{-1}$ in HBSS and stored frozen at −20°C in aliquots. Drugs used in the study are given in the Table and were stored at −20°C until used. Drug breakdown in storage was not determined but assumed to be minimal at this temperature and to be consistent between experiments.

*Collection, disaggregation and culture*:

All the tumours used were malignant astrocytomas (Kernohan grades III or IV) confirmed from paraffin wax section by Dr. D.I. Graham, Department of Neuropathology, Institute of Neurological Sciences, Southern General Hospital, Glasgow. Surgical biopsies were collected into the holding medium described above. Although satisfactory cultures were obtained even after 48 h storage at 4°C, the culture was usually initiated within 4 h of surgery. The sample was washed in DBSS, chopped into pieces of about 1–2 mm and washed twice more by resuspension and settling in DBSS. The pieces were finally suspended in 4.5 ml culture medium and transferred to a plastic culture flask (Falcon, 25 $cm^2$ growth area) at 20–50 pieces per flask. Collagenase (0.5 ml) was added to give a final concentration of 200 units $ml^{-1}$.

After 24–73 h in collagenase, disaggregation was completed by gentle pipetting and the cell suspension was centrifuged for 10 min at 500 g to remove the enzyme. The resulting pellet was resuspended in 5 ml fresh culture medium and transferred to a new flask. Depending on growth rate, viability and size of sample, a confluent monolayer took 1–3 weeks to form. The cells were then subcultured and used in drug experiments as secondary or tertiary cultures.

*Drug experiments*:

Monolayer cultures were trypsinised in exponential growth phase and 96–well microtitration plates (Nunclon or Linbro) were seeded to give $10^3$ cells per well. After 1–3 days, serial dilutions of the drugs (Table) in culture medium were added to the plates. One or two wells in each row were left free of drugs to act as controls. The plates were allowed to equilibrate with 2% $CO_2$ for 30 min and then sealed with Mylar film (Flow Laboratories) and replaced at 36.5°C for 3 days. Medium was removed by suction and the drugs replaced at 24 and 48 h. With most cell lines this corresponded to at least one population doubling time. In all drug experiments

**Table** Drugs used in the study

| *Chemical Name* | *Trade Name* | *Supplier* | *Highest Concentration used* ($mM\, l^{-1}$) |
|---|---|---|---|
| Bleomycin (BL) | — | Lundbeck | 0.0014 |
| CCNU | Lomustine | Lundbeck | 0.202 |
| Methyl CCNU (Me-CCNU) | Semustine | National Cancer Institute | 0.202 |
| Cytosine Arabinoside (Ara-C) | — | Sigma | 11.9 |
| 5-Fluoro-uracil (5-FU) | — | Sigma | 7.69 |
| Mustine HCl (MU) | Mustine | Boots | 17.3 |
| Procarbazine HCl (PCB) | Natulan | Roche | 4.5 |
| Vinblastine $SO_4$ (VB) | Velbe | Lilly | 0.123 |
| Vincristine (VCR) | Oncovin | Lilly | 0.0006 |
| VM26 | — | Sandoz | 0.02 |

cell counts were made of replicate plates of each cell line to determine population doubling time and to ensure that the cultures remained in exponential growth throughout drug treatment and recovery. In time course experiments, drug exposure periods of 1, 2 and 3 days were employed.

After drug exposure, the monolayer was washed 3× with fresh medium and the culture continued ("recovery period"). Throughout the recovery period the medium was replaced at least every 48 h. In time course experiments, a period of 3 h recovery was allowed for the equilibration of acid soluble pools and efflux of unbound drug when labelling was performed shortly after drug removal.

*Sampling by scintillation counting*

At intervals during drug exposure and recovery the growth medium was replaced with medium containing 20 $\mu$Ci ml$^{-1}$ L-leucine-4, 5-[$^3$H] (specific activity 10 mCi M$^{-1}$) and incubated for a further 4 h. The plates were washed, and cell protein, solubilized in 1N NaOH, counted as previously described (Freshney *et al.*, 1975). The [$^3$H] leucine incorporation of each well in a row was expressed as a percentage of the control in that row and the dose of drug which inhibited protein synthesis by 50% ($ID_{50}$) or 90% ($ID_{90}$) determined.

*Sampling by scintillation autofluorography*

In later experiments, uptake was determined by labelling the cultures with [$^{35}$S] methionine (2–20 $\mu$Ci ml$^{-1}$, specific activity 15–92 Ci M$^{-1}$, 218 Ci M$^{-1}$ before addition to medium) and adding scintillation fluid directly to the acid insoluble residues in the microtitration plate (Freshney & Morgan 1978). The solvent was dried off by centrifugation and autofluorograms were prepared on Kodak RP Royal-X X-ray film at −70°C. The autofluorograms were analysed densitometrically on a Helena Autoscanner with a 7×2 mm slit and $ID_{50}$ and $ID_{90}$ values determined as above.

*Cloning*

Exponentially-growing cells were treated with drugs in 25 cm$^2$ flasks, the drugs being replaced daily for 3 days. The flasks were then trypsinised and by determining the number of cells in the control flask each cell suspension was diluted to give 50–200 cells ml$^{-1}$.

The cells were plated out, either alone or on mitomycin-C treated (2 $\mu$g ml$^{-1}$ overnight) homologous feeder layers in 9 cm petri dishes (Nunclon). These were incubated for 2–3 weeks, fixed, stained and the colonies counted.

### Results

*Characterisation of cultures*

Twenty-four anaplastic astrocytomas, all of which grew well as monolayers, were selected for this study. Primary cultures of cells with long thin processes tended to form a network in sub-confluent cultures, but gave way to polygonal or spindle-shaped cells after serial passage. Although karyotypes were not performed on all the lines used, examination of some, and of many similar lines derived in the same manner, showed considerable aneuploidy with modal chromosome numbers ranging from 40–46 chromosomes (Guner *et al.*, 1977, Freshney, 1980). Recent evidence has shown that these cell cultures are capable of growth on confluent monolayers of normal contact-inhibited cells, while cells cultured from normal brain are not (Freshney *et al.*, 1980) and that they have a higher labelling index with [$^3$H] TdR at terminal cell density than cultures derived from normal brain (Morgan & Freshney, 1980).

*Derivation of sensitivity measurements*

Percentage inhibition curves displaying the effect of drug concentration on inhibition of [$^3$H] leucine incorporation for 5-fluorouracil (5FU), cytosine arabinoside (Ara-C), mustine (MU) and vinblastine (VB) were used to derive $ID_{50}$ values which were then plotted against time from addition of drug (Figure 1). Increasing the exposure time reduced the $ID_{50}$ and a further decrease was often observed during continued culture after removal of drug. In some cases, particularly with 5-FU, a plateau value was established between 6 and 8 days.

Standard deviations of 4 replicate cultures exposed to 5-FU and Ara-C were 20% and 16% of the mean respectively, at the time of drug removal, and 28% and 43% respectively, at the end of the assay.

It has been assumed that an exposure period of 3 days allows most of the proliferating cells to pass through the cell cycle at least once in the presence of drug. As cycle times are known to vary within one tumour cell population and between cell lines from different tumours, some cells will not have completed a full cycle in the presence of drugs. However, this is such a small proportion that adapting the assay to suit such extremes would be technically unmanageable.

*Comparison of $ID_{50}$ values derived from microtitration assay and clonogenicity*

A series of experiments was performed with 5 cell lines and 6 different drugs. The $ID_{50}$ values were determined in microtitration plates using

**Figure 1** Development of $ID_{50}$ with time. Astrocytoma cells were labelled with [$^3$H] leucine after drug treatment, incorporation determined by conventional scintillation counting and $ID_{50}$ values derived from % inhibition plotted against drug concentration. 5-fluorouracil (5-FU) (○); cytosine arabinoside (Ara-C) (●); vinblastine (VB) (▲); mustine (MU) (■). MU sensitivity was determined on IJK cells; 5-FU, Ara-C and VB on WMD cells.

scintillation autofluorography after 3 days drug exposure and 5 days recovery and by cloning the cells on an homologous mitomycin-C treated feeder layer immediately after 3 days drug exposure. The results (shown in Figure 2a) show a high degree of correlation between the 2 methods, the correlation coefficient being 0.96 ($p \leq 0.001$). Two of the points lying furthest off the regression line belong to GMS, a cell line found subsequently to contain a mixed population of endothelial and glial cells.

A comparison of $ID_{90}$ values by microtitration and cloning gave a lower correlation coefficient (0.82, $p \leq 0.001$) (Figure 2b). Although the slope of the regression line was similar, there was greater scatter and this was not confined to any one cell line.

When the 5 cell lines were ranked in order of sensitivity by cloning and microtitration, the only major discrepancy arose with GMS and VM26 where cloning gave a lower $ID_{50}$ than microtitration by 2 orders of magnitude. Any other alterations in ranking sequence were only due to minor differences in $ID_{50}$. Ranking the cell lines by $ID_{90}$ however, produced several discrepancies in keeping with the increased scatter observed in the regression plot.

*Comparison of $ID_{50}$ time courses in primary, secondary and tertiary cultures and in multiple cultures from the same biopsy*

Assays were performed on 2 cell lines (BRO and HNY) at different stages of culture (Figure 3). Microtitration plates were set up (a) directly after removal of collagenase (primary culture), (b) after trypsinisation of the primary culture (secondary culture), and (c) after trypsinisation of the secondary culture (tertiary culture). These cultures were treated with 5-FU and MU and their viability determined by [$^3$H] leucine incorporation and scintillation counting. Assays done on primary cultures showed marked oscillations in $ID_{50}$ with time and did not conform to the time course predicted by the HeLa cell model (Freshney *et al.*, 1975). However, the secondary and tertiary cultures behaved in a more predictable fashion.

A comparison of replicate cultures and multiple cultures from the same biopsy also revealed that the greatest variability occurred during the early stages of an assay and the possibility exists that there is some degree of parasynchrony of the cell cycle induced by transfer prior to the commencement of the assay. With 5-FU the early variability has gone by the time drugs are removed at 3 days, but with some other drugs, particularly vincristine and nitrosourea it may still persist.

*Differences between individual tumours*

A series of comparative experiments were performed, using tertiary cultures, to determine whether cultures from separate gliomas would exhibit differing sensitivities. Secondary cultures were treated with Ara C, 5-FU, VB and MU and viability was determined by labelling with [$^3$H] leucine followed by conventional scintillation counting. There were 5 main features of these curves (Figure 4 a–d):

**Figure 2** Correlation of microtitration and clonogenic survival curves. Microtitration plates were labelled with [$^{35}$S] methionine, 5 days after removal of drugs, incorporation measured by autofluorography and $ID_{50}$ (a) and $ID_{90}$ (b) values derived from the survival curves. Correlation coefficients were 0.96 and 0.82 for (a) and (b) respectively. In (a) the broken line represents the regression with G-GMS points excluded. (For Figure 2b see overpage).

1) The $ID_{50}$ in all cases fell rapidly, although not always continuously, between 24 and 72 h.
2) A low $ID_{50}$ at 24 h was often, but not invariably, accompanied by a steep gradient in the rate of change of $ID_{50}$.
3) A plateau of maximum sensitivity was reached with 5-FU at 72 h.
4) Vinblastine showed the greatest differences in sensitivity between lines suggesting a division into 2 groups, one insensitive and the other so sensitive that the $ID_{50}$ drops below the minimum concentration.

 5-FU sensitivities were similar by the end of the assay with the exception of one line that was apparently resistant. This line had a long doubling time of 132 h. In most cases, however, a steep slope or low 3-day $ID_{50}$ value did not correlate with a short doubling time.
5) Divergence in the curves for Ara-C suggested that there may have been differences in the rates at which maximum sensitivity was reached, but by the time maximum sensitivity was reached the differences in $ID_{50}$ had diminished. Extrapolation might suggest further convergence had the assay continued for longer.

*Extended series of comparative observations*

A more extensive series of observations was made with a different group of 20 tumours using the drugs procarbazine (PCB), CCNU, methyl-CCNU (Me-CCNU) and vincristine (VCR). These 4 drugs are amongst those most frequently used clinically for human glioma and are among the most effective. The results of this series are shown in Figure 5 (a–c) condensed to give the $ID_{50}$ values after a 24–72 h recovery period at the end of the log phase of growth, a point reached at different times by different cultures. Four observations can be made.

1) The sensitivity to PCB was low; no $ID_{50}$ fell below the range of the predicted plasma level, although 3/13 (30%) fell within it.
2) All of the $ID_{50}$ values for VCR fell below the predicted plasma concentration. Ten of 16 were closely grouped between $10^{-10}$ M and $2\times10^{-9}$ M, while the remaining 6 (38%) showed

**Figure 3** Development of $ID_{50}$ values from primary (X); secondary (○); tertiary (●) cultures of 2 cell lines (BRO and HNY). Viability was determined by [$^3$H] leucine incorporation determined by scintillation counting.

higher sensitivities between $10^{-14}$ M and $10^{-11}$ M. Time course data (not shown) suggested that these six $ID_{50}$ values were stable.

3) Although 4/13 (31%) of nitrosourea estimations fell below the predicted plasma level, the majority registered no measurable $ID_{50}$ by the end of the assay. Two of the 4 samples which did not show a measurable $ID_{50}$ had been sampled earlier than the others because cell proliferation was approaching density limitation. An extrapolation of the time course of the development of sensitivity (data not shown) indicated $ID_{50}$ values for these 2 examples, above the threshold of the assay 3 days after drug removal. This effectively reduces the number of sensitive samples to 2/13 (15%).

**Figure 4** Time courses of the development of sensitivity to ara-C (a), 5-FU (b). VB (c), and MU (d) by 15 different early passage cell lines derived from different astrocytomas. Viability was determined by [$^3$H] incorporation and conventional scintillation counting. Arrows indicate points which fall below axes.

4) Five cultures giving $ID_{50}$ values for nitrosourea above the threshold by the end of the assay had shown low $ID_{50}$ values ($10^{-5}$–$10^{-7}$ M) immediately after exposure to the drug (data not shown). This suggests that the effect of the drug on these cultures was transitory and resistance was readily achieved.

### Discussion

The examination of replicate cultures suggests that the internal variation of the microtitration assay can be minimised by sampling 3–5 days after drug removal. With most cell lines this allows for at least one complete cell cycle in the absence of drugs, given that the average population doubling time for these cultures is about 36 h and the cell cycle time of each line will vary around that figure. It was shown previously with HeLa cells (Freshney, 1976) that prolonged exposure to drugs and recovery were necessary to obtain a measurable and stable $ID_{50}$ value for many drugs particularly those which are phase-specific. Present results indicate that stable $ID_{50}$ values for several drugs can be demonstrated with cultures from human glioma, but

**Figure 5** Sensitivities of 20 different astrocytomas to PCB (a), VCR (b) and a nitrosourea (c). Viability was determined by [$^{35}$S] methionine incorporation followed by autofluorography and $ID_{50}$ values were determined densitometrically. Each point represents the terminal $ID_{50}$ values measured at the end of the recovery period. Solid symbols in (c) are Me CCNU and open symbols CCNU. The bars indicate approximate peak plasma levels attainable in man are taken from published data by Alberts & Chen (1980) VCR and nitrosoureas (using values for BCNU) and Schwartz & Kessler (1967 unpublished) for PCB.

that many samples are still resistant particularly to nitrosoureas, even after prolonged exposure and recovery (see below). There is no evidence for differences in the sensitivity of multiple cultures from different parts of the same biopsy. This does not mean that differences do not exist but that variant populations must represent a small component of the total population.

The selection of a microtitration plate assay for this series of measurements was influenced by difficulties in obtaining reasonable plating efficiencies for a clonogenic assay. However, the selection of the appropriate $CO_2$ tension (2%), use of glucocorticoids in the medium (Guner *et al.*, 1977) and cloning on homologous feeder layers (Freshney *et al.*, 1980) have enabled cloning efficiencies from 5%–20% to be achieved, making a clonogenic assay more feasible. Even though the 2 assays have been shown to agree, the microtitration assay is quicker and more adaptable to automated handling and analysis. Although correlation of the $ID_{50}$ values is very good some discrepancies between microtitration and cloning can be seen when $ID_{90}$ values are compared. This may reflect the greater sensitivity of the clonogenic assay in detecting a small resistant fraction ($\leq 5\%$) which would not be detectable in the microtitration assay using autofluorography. Nevertheless, the ability of the microtitration method to process a large number of samples in a semi-automated manner and provide gross sensitivity data comparable to clonogenic survival curves, makes it a very valuable technique for rapid screening. The interpretation of a clonogenic assay is usually straightforward as it is based on counting surviving colonies of cells. To be equivalent the $ID_{50}$ at the end of the microtitration assay should be used and the assay continued for the maximum culture period possible before density limitation of growth is apparent.

With the microtitration assay, cultures are usually available for testing within 3 weeks of surgery and with an assay taking 10–14 days to perform, a result is available within 4–5 weeks of operation. Currently patients who are to receive chemotherapy for cerebral glioma at the National Hospital do so after completion of radiotherapy—usually a minimum of 7–10 weeks after operation. Using a cloning assay where cells are treated in secondary culture with drugs for 3 days and then cloned, it might be difficult to obtain a result within 6–7 weeks of operation.

The acceptance of one parameter rather than another to predict drug sensitivity *in vivo* will be governed by which value correlates best with the clinical outcome. Preliminary data on a small number of patients suggest that there is an interesting correlation between *in vitro* data and clinical response (Darling & Thomas, 1981). In 6 patients with malignant glioma treated with PCB, CCNU and VCR following radiotherapy, three responded. Two of these patients responded *in vitro* to all 3 drugs and 1 responded to 2 of the 3 drugs. Of the 3 patients who failed to respond clinically, only 1 had a significant response *in vitro*. This work will be reported fully later.

The majority of samples treated with nitrosourea, VCR or PCB were insensitive. This is also apparent in the clinical treatment of glioma since varying degrees of remission still invariably give way to relapse. There is an indication of low frequency response rate in these studies (15% for VCR, 20% for PCB and 7% for nitrosourea) which, though difficult to evaluate statistically, is of the correct order of magnitude for the anticipated clinical response rate to chemotherapy. Perhaps the most interesting drugs in this survey were the Vinca alkaloids, since they alone produced persistent sensitivity, the sensitive samples falling into a separate and readily distinguishable group. This would perhaps be the best drug to test for clinical correlation. Sensitivity to PCB and nitrosourea was generally poor and showed little evidence of a permanent effect with most cultures. The low levels of sensitivity to PCB may have been due to the lack of generation of the more active metabolites produced *in vivo*. The nitrosoureas do not normally require metabolic activation. The poor response encountered with them may relate to the observation that high density non-cycling populations are often more sensitive to nitrosourea (Barranco *et al.*, 1973). Limitations in the microtitration assay makes this difficult to assess but attempts are being made to investigate this with the clonogenic assay.

It is unfortunate that some of the more effective drugs in this series of experiments, e.g. 5-FU, BL and Ara-C have not been found clinically useful. Presumably this reflects pharmacokinetic problems, principally access to tumour sites in the brain.

D.M. and R.I.F. are grateful to the Medical Research Council and the Cancer Research Campaign for financial assistance. J.L.D. and D.G.T.T. are grateful to the Brain Research Trust and the Cancer Research Campaign for financial assistance. The authors are also grateful to Dr. D. Graham of the Southern General Hospital, Glasgow for supplying tumour biopsies and for determining tumour histology and Mr. D. Robson, Roche Products Ltd for supplying details of the blood levels of procarbazine.

**References**

ALBERTS, D.S. & CHEN, H-S. G. (1980). Tabular summary of pharmacokinetic parameters relevant to *in vitro* drug assay. In *Cloning of human tumour stem cells.* (Ed. Salmon), New York: Alan R. Liss p. 351.

BARRANCO, S.C., NOVAK, J.K. & HUMPHREY, R.M. (1973). Response of mammalian cells following treatment with bleomycin and 1,3-Bis (2-chloroethyl) - 1-nitrosourea during plateau phase. *Cancer Res.*, **33,** 691.

DARLING, J.L. & THOMAS, D.G.T. (1981). Evaluation of the *in vitro* response of human gliomas to chemotherapeutic agents and its correlation with clinical response. *Br. J. Cancer,* **43,** 734.

FRESHNEY, R.I. (1976). Some observations on assay of anti-cancer drugs in culture. In *Human Tumours in Short Term Culture—Techniques and Clinical Applications.* (Ed. Dendy), London: Academic Press p. 150.

FRESHNEY, R.I. (1980). Tissue culture of glioma of the brain. In *Brain Tumours—Scientific Basis, Clinical Investigation and Current Therapy.* (Eds. Thomas & Graham) London: Butterworths. p. 21.

FRESHNEY, R.I. & MORGAN, D. (1978). Radioisotopic quantitation in microtitration plates by an autofluorographic method. *Cell Biol. Int. Rep.*, **2,** 375.

FRESHNEY, R.I., PAUL, J. & KANE, I.M. (1975). Assay of anti-cancer drugs in tissue culture; conditions affecting their ability to incorporate $^3$H-Leucine after drug treatment. *Br. J. Cancer,* **31,** 89.

FRESHNEY, R.I., SHERRY, A., HASSANZADAH, M., FRESHNEY, M., CRILLY, P. & MORGAN, D. (1980). Control of cell proliferation in human glioma by glucocorticoids. *Br. J. Cancer,* **41,** 857.

GUNER, M., FRESHNEY, R.I., MORGAN, D., FRESHNEY, M.G., THOMAS, D.G.T. & GRAHAM, D.I. (1977). Effects of dexamethasone and betamethasone on *in vitro* cultures from human astrocytoma. *Br. J. Cancer,* **35,** 439.

KNOCK, F.E., GALT, R.M., OESTER, Y.T. & SYLVESTER, R. (1974). *In vitro* estimate of sensitivity of individual human tumours to antitumour agents. *Oncology,* **30,** 1.

LIMBURG, H. & HECKMAN, U. (1978). Chemotherapy in the treatment of advanced pelvic malignant disease with special reference to ovarian cancer. *J. Obstet. Gynaecol.* (Br. Commonw.), **75,** 1246.

MORGAN, D. & FRESHNEY, R.I. (1979). *In vitro* predictive testing of astrocytoma. *Br. J. Cancer,* **40,** 203.

MORGAN, D. & FRESHNEY, R.I. (1980). Dexamethasone effect on glial cell surface glycoproteins. *Cell Biol. Int. Rep.,* **4,** 756.

NISSEN, E., TANNEBERGER, S., PROJAN, A., MORACK, G. & PEEK, U. (1978). Recent results of *in vitro* drug prediction in human tumour chemotherapy. *Arch. Geschwulstforsch,* **48,** 667.

SALMON, S.E., HAMBURGER, A.W., SOEHNLEN, B.S., DURIE, B.G.M., ALBERTS, D.S. & MOON, T.E. (1978). Quantitation of differential sensitivity of human-tumour stem cells to anti-cancer drugs. *N. Engl. J. Med.,* **298.** 1321.

THOMAS, D.G.T., DARLING, J.L., FRESHNEY, R.I. & MORGAN, D. (1979). *In vitro* chemosensitivity assay of human glioma by scintillation autofluography. In *Multidisciplinary Aspects of Brain Tumour Therapy,* (Eds. Paoletti *et al.*), Amsterdam: Elsevier North-Holland Biomedical Press. p. 19.